# High prevalence of syndemic health problems in patients seeking post-exposure prophylaxis for sexual exposures to HIV

**DOI:** 10.1371/journal.pone.0197998

**Published:** 2018-05-23

**Authors:** Steven A. Morrison, Deborah Yoong, Trevor A. Hart, Paul MacPherson, Isaac Bogoch, Vishalini Sivarajah, Kevin Gough, Mark Naccarato, Darrell H. S. Tan

**Affiliations:** 1 Division of Infectious Diseases, St. Michael’s Hospital, Toronto, Canada; 2 Department of Pharmacy, St. Michael’s Hospital, Toronto, Canada; 3 Department of Psychology, Ryerson University, Toronto, Canada; 4 Dalla Lana School of Public Health, University of Toronto, Toronto, Canada; 5 Division of Infectious Diseases, The Ottawa Hospital, Ottawa, Canada; 6 Division of Infectious Diseases, Toronto General Hospital, Toronto, Canada; 7 Department of Medicine, University of Toronto, Toronto, Canada; 8 Centre for Urban Health Solutions, St. Michael’s Hospital, Toronto, Canada; Oregon State University, UNITED STATES

## Abstract

**Introduction:**

The standard clinical approach to non-occupational HIV post-exposure prophylaxis (nPEP) focuses on biomedical aspects of the intervention, but may overlook co-occurring or ‘syndemic’ psychosocial problems that reinforce future vulnerability to HIV. We therefore sought to determine the prevalence of syndemic health problems in a cohort of Ontario nPEP patients, and explored the relationship between syndemic burden and HIV risk.

**Methods:**

Between 07/2013-08/2016, we distributed a self-administered questionnaire to patients presenting to three clinics in Toronto and Ottawa seeking nPEP for sexual HIV exposures. We used validated screening tools to estimate the prevalence of depression (CES-D score ≥16), harmful alcohol use (AUDIT ≥8), problematic drug use (DUDIT ≥6 men/≥2 women), and sexual compulsivity (SCS ≥24) among men who have sex with men (MSM) respondents. In exploratory analyses, we examined the relationships between syndemic conditions using univariable logistic regression models, and the relationship between syndemic count (total number of syndemic conditions per participant) and HIV risk, as estimated by the HIRI-MSM score, using linear regression models.

**Results:**

The 186 MSM included in the analysis had median age 31 (IQR = 26–36), including 87.6% having a college/undergraduate degree or higher. Overall, 53.8% screened positive for depression, 34.4% for harmful alcohol use, 30.1% for problematic drug use, and 16.1% for sexual compulsivity. Most participants (74.2%) had at least one syndemic condition and 46.8% had more than one. Exploratory analyses suggested positive associations between depression and harmful alcohol use (OR = 2.11, 95%CI = 1.13, 3.94) and between harmful alcohol use and problematic drug use (OR = 1.22, 95%CI = 0.65, 2.29). Syndemic count was associated with increased HIRI-MSM risk scores in univariable (2.2, 95%CI = 1.0, 3.3 per syndemic condition) and multivariable (2.1, 95%CI = 0.6, 3.6) linear regression models.

**Conclusions:**

The prevalence of syndemic conditions in MSM seeking nPEP for sexual exposure is alarmingly high, and is associated with underlying HIV risk. Routine screening for these conditions may identify opportunities for intervention and could alleviate future vulnerability to HIV.

## Introduction

HIV post-exposure prophylaxis (PEP), involving 28 days of antiretroviral medications after an HIV exposure, is an effective prevention strategy that has become the standard of care in North America in both occupational settings such as needlestick injuries (oPEP), and non-occupational settings such as condomless sexual activity (nPEP).[1, 2] Traditionally, the focus of nPEP delivery has been on biomedical aspects of the intervention including clinical assessment, sexually transmitted infection (STI) screening, coordinating medication access and monitoring for side effects. Notably, this approach tends to overlook important co-existing conditions that may underlie HIV risk behaviour.[3] Many individuals seeking nPEP come from populations at high ongoing HIV risk, particularly gay, bisexual and other men who have sex with men (MSM).[4] In Canada, MSM continue to bear a grossly disproportionate burden of incident HIV infections, with an estimated HIV risk that is 131 times higher than in other Canadian men, at 469 versus 3.6 infections per 100,000 persons.[5] A rich literature has previously documented the high burden of co-existing psychosocial and mental health conditions in MSM, including substance use and depression (reviewed in [6–8]). These health concerns, and related problems such as sexual compulsivity, are often referred to as ‘syndemic’ conditions in the literature on HIV prevention, since they are interconnected, co-endemic health problems that are mutually reinforcing.[9, 10]

Syndemic theory posits that when multiple epidemics co-occur and interact synergistically, there is an increase in the burden of disease within the population.[10] Multiple studies have identified co-occurring and/or mutually reinforcing syndemic conditions that predispose to HIV infection.[11–15] For example, a longitudinal study of 4,295 HIV negative MSM found a positive dose-response relationship between the syndemic ‘count’, defined as the total number of syndemic conditions present in an individual (including depressive symptoms, heavy alcohol use, stimulant use, polydrug use, and childhood sexual abuse) and HIV incidence over time.[16] Understanding the frequency of and relationships between such issues among nPEP patients is important, as it may uncover additional clinical priorities that warrant attention during nPEP patient encounters such as referrals to mental health and addictions services.

To our knowledge, only a single study has previously examined the burden of syndemic conditions in nPEP patients, and found a high prevalence of pre-existing mental health diagnoses through retrospective case review.[17] We therefore sought to prospectively estimate the prevalence of depression, harmful alcohol use, problematic drug use, and sexual compulsivity in patients seeking nPEP at our institutions. We hypothesized that these syndemic conditions would be common, and reasoned that documenting a high prevalence would support efforts to systematically screen for them in the future. Our secondary objective was to assess respondent attitudes and experiences of discrimination relevant to HIV risk behaviours, to identify potential topic areas for clinicians to explore during risk-reduction counseling. Exploratory objectives were to examine the co-occurrence of the syndemic conditions, to assess for a relationship between syndemic count and overall HIV risk, and to explore whether such a relationship might be additive or synergistic in nature.

## Methods

### Study design

We distributed a one-time self-administered questionnaire to patients seeking nPEP between July 2013 and August 2016, and reviewed patient charts six months post-enrolment. Any adult presenting for the first time to participating infectious diseases clinics at St. Michael’s Hospital, Toronto General Hospital, or The Ottawa Hospital regarding nPEP for potential sexual exposures to HIV was eligible to complete the questionnaire. Because the vast majority of participants were MSM, we restricted our analyses to this population.

Patients were approached sequentially to minimize potential selection bias. All participants were offered a Can$15 honorarium. The questionnaire was preceded by a detailed letter of information about the study. We used an implied consent process in which completion of the questionnaire was considered consent to participate. This study was approved by the Research Ethics Boards of St. Michael's Hospital, The Ottawa Health Science Network, and the University Health Network.

### Measures

The study instrument included questions pertaining to demographics and sexual activity, in addition to several previously published, validated screening tools. To objectively quantify HIV risk, we used the HIV Incidence Risk Index for MSM (HIRI-MSM), a screening tool for HIV risk developed by the Centers for Disease Control and Prevention. The recommended cutoff score to identify men as high risk is ≥10, conferring a sensitivity of 84% and specificity of 45% for predicting incident HIV infection in the following six months.[18] Questionnaires were self-completed by participants in private, to minimize social desirability bias.

Our primary objective was to estimate the prevalence of depression, harmful alcohol use, problematic drug use, and sexual compulsivity in the study cohort, using the following standardized scales and cutoff values. On the 20-item Center for Epidemiologic Studies-Depression (CES-D) scale,[19] we used a cut-off score of 16, which has been established as a useful screening tool for depression (Cronbach’s α = 0.92).[20, 21] On the 10–item Alcohol Use Disorder Identification Test (AUDIT),[22] we used a cutoff score of eight, which gives a sensitivity and specificity as good as, or superior to comparator scales for harmful alcohol use (α = 0.87).[23] On the 11–item Drug Use Disorder Identification test (DUDIT),[24] we used a cutoff of six, which in men, indicates drug-related problems (α = 0.88).[24] On the 10–item sexual compulsivity scale (SCS),[25, 26] we used the recommended cutoff of 24 to indicate problems with ‘sexual addiction’ (α = 0.93).[26, 27]

Our secondary objectives were to assess attitudes relevant to HIV risk behaviours. These included perceptions of HIV risk, which we measured using the 16-item Disengagement Coping with HIV Risk (DCHR) scale (α = 0.86).[28] Its three sub-scales measure fatalistic beliefs about eventually acquiring HIV (‘HIV fatalism’, eg. “I sometimes wonder if it’s worth all the trouble it takes to stay HIV-negative”), reduced perceived severity of HIV infection due to medical advances (‘HIV optimism’, eg. “I’m less concerned about getting AIDS now that there are new effective medicines to treat it”), and negative affective states associated with the risk of HIV infection (‘anxieties’, eg. “I give myself grief about not protecting myself”). We used the 14-item Multi-Axial gay men’s Inventory–Men’s Short Version (MAGI-MSV) to assess four domains of internalized homophobia: low self-esteem related to sexual orientation (eg. “Whenever I think about being gay, I feel depressed”, discomfort with public appearances related to homosexuality (eg. “Some gay men are too effeminate”), maladaptive responses to homosexuality (eg. “Over the past 2 years, I have actually attempted suicide because I could not accept my homosexuality”, and negative feelings towards homosexuality due to HIV/AIDS (eg. “Occasionally, when I think about AIDS, I start wishing that I weren’t gay”) (α = 0.92).[29] We used the 9-item Benefits of Barebacking scale (BBS) to measure perceived benefits of intentional condomless anal intercourse among MSM (eg. “Barebacking is sexier than sex with condoms” α = 0.92).[30] We also included the 9-item Sexual Sensation Seeking (SSS) Scale,[25] to explore motivations for engaging in riskier sexual activity (eg. “I like new and exciting sexual experiences and sensations”, α = 0.80). For all four attitude scales, lower scores indicate lower levels of the measured construct. Finally, to assess MSM participants’ experiences of homophobia, we included two four-level Likert scale items of our own design, reading, “I have experienced discrimination related to my sexual orientation in my life” and “I experience discrimination related to my sexual orientation often”.

### Chart review

During the study period, clinical nPEP protocols at the participating sites recommended a 28-day regimen of tenofovir disoproxil fumarate/emtricitabine with either lopinavir/ritonavir or raltegravir, although the timing of final follow-up visits changed from 4–6 months to 3 months post-exposure during the course of the study. We reviewed patient charts six months post-enrolment to assess clinical outcomes, including prevalent STIs at the time of presentation, HIV status, nPEP side effects, and completion of follow-up.

### Statistical analyses

Primary and secondary analyses for this study were descriptive. We tabulated scores for each of the included scales and sub-scales for each respondent, and summarized results using both actual scores as continuous outcomes, and proportions of participants surpassing cutoffs as dichotomous outcomes. Results are presented as medians (interquartile ranges) and frequencies (percentages) as appropriate.

In exploratory analyses, we first used logistic regression models to examine the bivariable relationships between depression, harmful alcohol use, problematic drug use and sexual compulsivity (reporting the results using odds ratios with 95% confidence intervals), anticipating positive associations between these closely related conditions. Next, to examine the hypothesis that syndemic burden is associated with the degree of HIV risk among MSM, we constructed simple and multivariable linear regression models using the syndemic count (defined as the total number of syndemic conditions present in each participant) as the primary predictor variable and the HIRI-MSM score as the outcome variable. When building the multivariable model, we considered all available demographic, clinical, attitude- and discrimination-related data, after removal of variables due to collinearity and exclusion of variables that are components of the HIRI-MSM index (ie. age and the type of sexual exposure prompting nPEP use). Patient characteristics were retained as covariates in the final model if they changed the magnitude of the parameter estimate for the primary predictor variable by ≥10% in bivariable analyses. Results are reported as beta estimates with 95% confidence intervals and p-values. Finally, applying an approach suggested by other authors to ascertain whether this relationship might be additive or synergistic in nature,[31] we constructed a separate multivariable linear regression model in which each syndemic condition, as well as each possible 2-way, 3-way and 4-way interaction term between these conditions, was included as a predictor variable, and the HIRI-MSM score was included as the outcome variable.

Missing data were excluded from the analyses. All statistical analyses were performed between 2016 and 2017 using SAS® version 9.4 (SAS® Institute, NC).

### Sample size

The target sample size was based on the number of participants required to determine the prevalence of syndemic health problems in the sample with reasonable precision. Using the equation N = (Z_1-α/2_)^2^ * p(1-p) / *l*^2^, where Z_1-α/2_ is the 1-α/2 critical value of the standard normal distribution, p is the proportion of interest, *l* is half the length of the desired 95% confidence interval, and N is the required sample size, a conservative estimate of 50% prevalence determined that 171 participants were needed to achieve a 95% confidence interval that was 15% wide.

## Results

Of 375 eligible patients who were seen in clinic on recruitment days, 203 agreed to participate, giving acceptance rates of 173/314 (55.1%), 25/49 (51.0%) and 5/12 (41.7%) at the three sites respectively. The overwhelming majority of respondents (186/203, 91.6%) were MSM based on their reported sexual activity (n = 5) and/or self-identifying as gay, bisexual or queer (n = 181); hence all further analyses were restricted to this group. Characteristics of the 186 participants are reported in Table 1. Enrolment varied considerably between sites due to differences in staff availability, participant demographics were broadly similar. Median age was 31 (26–36) and about half of respondents were White (48.9%, 85/174), with most having a college/undergraduate degree or higher (87.6%, 163/186). Over half of participants presented for condomless anal receptive intercourse (55.9%, 104/186) and perceived themselves to be at no/low (55.2%, 90/163) as opposed to moderate/high overall HIV risk. Of the 165 participants tested, 8.5% had an STI at baseline, including gonorrhea (3.2%, 5/157), chlamydia (3.2%, 5/156) and syphilis (3.5%, 4/113).

**Table 1:** Participant characteristics (n = 186 MSM)^a^.

Characteristic	Value^b^
Age–median (IQR)	31 (26, 36)
Clinical site	
St. Michael’s Hospital	160 (86.0)
The Ottawa Hospital	21 (11.3)
Toronto General Hospital	5 (2.7)
Education–frequency (%)	
High school diploma or less	23 (12.4)
College or undergraduate degree	91 (48.9)
Graduate or professional degree	72 (38.7)
Ethnicity–frequency (%)	
White	85 (48.9)
Non-White	89 (51.2)
Perceived HIV risk–frequency (%)	
No/low risk	90 (55.2)
Moderate/high risk	75 (44.8)
Medication coverage–frequency (%)	
Private insurance	64 (43.5)
Other	83 (56.5)
Positive baseline diagnostic tests–frequency (%)	
Syphilis	4 (3.5)
Hepatitis B	0 (0.0)
Hepatitis C	1 (0.6)
Chlamydia (any site)	5 (3.2)
Gonorrhea (any)	5 (3.2)
Any bacterial sexually transmitted infection	14 (8.5)
Highest risk type of incident sexual exposure–frequency (%)	
Condomless receptive anal	104 (55.9)
Condomless insertive anal	82 (44.1)
Sexual behavior in past six months–median (IQR)	
Number of male sexual partners	6 (3, 11)
HIV+ partners	0 (0, 1)
HIV status of partner unknown	1 (0, 5)
Number of condomless sexual encounters with HIV+ partner	
Anal receptive	0 (0, 0)
Anal insertive	0 (0, 0)
Number of condomless sexual encounters with HIV- partner	
Anal receptive	0 (0, 1)
Anal insertive	0 (0, 1)
Number of condomless sexual encounters with unknown status partner	
Anal receptive	0 (0, 1)
Anal insertive	0 (0, 1)
HIRI-MSM score–median (IQR)	18 (12, 22)
HIRI-MSM score ≥ 10 –frequency (%)	150 (80.7)
Discrimination related to sexual orientation–lifetime	
Strongly agree / agree	105 (58.7)
Strongly disagree / disagree	74 (41.3)
Discrimination related to sexual orientation—often	
Strongly agree / agree	36 (20.1)
Strongly disagree / disagree	143 (79.9)
nPEP regimen completion–frequency (%)	
Yes	98 (52.7)
No	9 (4.8)
Unknown	79 (42.5)
HIV positive at 3 months–frequency (%)	1 (0.5)

^a^ MSM, Men Who Have Sex with Men; HIRI-MSM, HIV Incidence Risk Index for Men Who Have Sex with Men; nPEP, non-occupational Post-Exposure Prophylaxis

^b^ Values represent medians (interquartile range) or frequencies (percentages). All percentages calculated out of totals with available data

The median number of male partners in the past six months was six (IQR = 3–11), with 72.0% (118/164) of participants reporting at least one sexual encounter with a partner of unknown HIV status and 42.4% (75/177) with a partner of known HIV-positive status. The median score on the HIRI-MSM scale was 18 (IQR = 12–22) and 80.7% (150/186) met the cutoff for high objective HIV risk. Of note, these figures likely underestimate overall HIV risk since 33.3% (62/186) had at least one missing response in the HIRI-MSM scale.

The prevalence of syndemic conditions within the study cohort is described in Table 2. Over half of participants (53.8%, 100/186) screened positive for depression on the CES-D. Harmful alcohol use and problematic drug use were also common, with 34.4% (64/186) screening positive on the AUDIT and 30.1% (56/186) on the DUDIT. Sexual compulsivity was seen in 16.1% (30/186) of respondents. At least one syndemic condition was present in 74.2% (138/186) of the sample and 46.8% (87/186) had more than one. The median syndemic count was 1 (0, 2).

**Table 2:** Syndemic conditions and attitude scales in study cohort (n = 186 MSM).

Screening test	Result	Scale range^a^
CES-D scale		
Score ≥16 –no. (%)	100 (53.8)	-
Median score (IQR)	18.0 (10.0, 27.0)	0–60
AUDIT		
Score ≥8 –no. (%)	64 (34.4)	-
Median score (IQR)	6.0 (3.0, 9.0)	0–40
DUDIT		
Score ≥6 –no. (%)	56 (30.1)	-
Median score (IQR)	4.0 (2.0, 9.0)	0–44
SCS		
Score ≥24 –no. (%)	30 (16.1)	-
Median score (IQR)	15.0 (12.0, 19.0)	10–40
Number of syndemic conditions–no. (%)		
Zero	48 (25.8)	-
One	51 (27.4)	-
Two	65 (35.0)	-
Three	19 (10.2)	-
Four	3 (1.6)	-
DCHR Scale–median (IQR)	29.0 (24.0, 37.0)	
HIV fatalism	7.0 (6.0, 9.0)	6–30
HIV optimism	9.0 (6.0, 14.0)	6–30
Anxieties	13.0 (9.0, 15.0)	4–20
MAGI-MSV scale–median (IQR)^b^	6.5 (2.0, 13.0)	0–42
Gay self-assurance and worth	2.0 (0.0, 7.0)	0–21
Public appearance of homosexuality	2.0 (0.0, 5.0)	0–9
Extreme/maladaptive measures to homosexuality	0.0 (0.0, 0.0)	0–6
Impact of HIV/AIDS on homosexuality	0.0 (0.0, 2.0)	0–6
BBS–median score (IQR)	20.0 (13.0, 28.0)	9–45
SSS scale–median (IQR)	20.0 (16.0, 24.0)	9–36

^a^ Refer to Methods section for additional scale range information

^b^ This scale is designed for gay/MSM populations and was not completed by participants identifying as non-MSM.

CES-D, Center for Epidemiologic Studies-Depression; AUDIT, Alcohol Use Disorder Identification Test; DUDIT, Drug Use Disorder Identification test; SCS, Sexual Compulsivity Scale; SSS, Sexual Sensation Seeking; DCHR, Disengagement Coping with HIV Risk; MAGI-MSV, Multi-Axial Gay Men’s Inventory—Men’s Short Version; BBS, Benefits of Barebacking scale

Median scores for the attitude scales are reported in Table 2. The median DCHR subscale scores showed low levels of HIV fatalism (7.0, IQR = 6.0–9.0) and optimism (8.0, IQR = 6.0, 14.0), and moderate levels of HIV-related anxiety (13.0, IQR = 10.0–15.0), compared to the ranges of possible scores (6–30, 6–30, 4–20 respectively). The median MAGI-MSV score (6.5, IQR = 2.0–13.0) indicated low levels of internalized homophobia, as further reflected in each subscale: Gay self-assurance and worth (2.0, IQR = 0.0–7.0), Public appearance of homosexuality (2.0, IQR = 0.0–5.0), Extreme/maladaptive measures to homosexuality (0.0, IQR = 0.0–0.0), and Impact of HIV/AIDS on homosexuality (0.0, IQR = 0.0–2.0). The relatively low median score on the BBS (20.0, IQR = 13.0–28.0) indicates that, overall, MSM did not perceive strong benefits to condomless anal intercourse. The median score on the SSS scale was moderate (19.5, IQR = 16.0–23.0), indicating that most participants only partially identified as sexual sensation-seeking.

### Clinical outcomes

The six-month chart review revealed that among participants with available data, 91.6% (98/107) completed the entire nPEP regimen, and 36.0% of all patients (67/186) attended at least one follow-up appointment three months post-enrolment or later. One individual tested HIV positive at baseline, only 16 hours post-exposure, indicating seroconversion resulted from a previous exposure. The other 98.4% (61/62) of participants tested at 12 weeks post-exposure remained HIV negative.

### Exploratory objectives

Unadjusted logistic regression analyses suggested positive associations between the four syndemic conditions (Table 3), although the only relationships that reached statistical significance were between depression and harmful alcohol use (OR = 2.11, 95%CI = 1.13, 3.94) and between harmful alcohol use and problematic drug use (OR = 1.22, 95%CI = 0.65, 2.29).

**Table 3:** Unadjusted odds ratios (95% confidence intervals) quantifying the bivariable relationships between four syndemic conditions (n = 186 MSM).

Syndemic condition	Harmful alcohol use	Drug-related problem	Sexual addiction
**Depression**	2.11 (1.13, 3.94)	1.22 (0.65, 2.29)	1.35 (0.61, 3.00)
**Harmful alcohol use**		2.10 (1.10, 4.01)	1.13 (0.50, 2.54)
**Drug-related problem**			1.43 (0.63, 3.24)
**Sexual addiction**			

In linear regression models exploring the relationship between syndemic count and HIV risk, we observed an estimated 2.2-point increase (95%CI = 1.0, 3.3) in the HIRI-MSM score per syndemic condition in the unadjusted analysis (Table 4). Younger age and presenting for nPEP due to condomless receptive anal sex were also positively associated with higher risk, as would be expected since both these variables are incorporated into the HIRI-MSM score. Other variables associated with a higher risk score in unadjusted analyses included, moderate/high perceived HIV risk, having a bacterial STI at baseline, lifetime experience of homophobia, and higher scores on the DCHR, BBB and SSS scales. The relationship between syndemic count and HIV risk persisted in the multivariable model, with an estimated 1.6-point HIRI-MSM score increase (95%CI = 0.1, 3.1) per syndemic condition, with moderate/high perceived HIV risk and lifetime experience of homophobia also retaining a statistically significant association with higher risk scores.

**Table 4:** Linear regression estimates of the associations between participant characteristics and HIRI-MSM scores (n = 186 MSM).

Predictor variable	Univariable		Multivariable	
	Beta estimate (95% CI)	p-value	Beta estimate (95% CI)	p-value
Syndemic count	**2.2 (1.0, 3.3)**	**0.0002**	**1.6 (0.1, 3.1)**	**0.03**
Age (per decade increase)	**-1.9 (-3.1, -0.6)**	**0.004**		
Education				
High school diploma or less	Ref			
College or undergraduate degree	1.8 (-2.0, 5.6)	0.36		
Graduate or professional degree	1.1 (-2.8, 5.0)	0.57		
Ethnicity				
White	Ref		Ref	
Nonwhite	1.9 (-0.6, 4.4)	0.14	1.9 (-0.9, 4.7)	0.18
Perceived HIV risk				
No/Low risk	Ref		Ref	
Moderate/High risk	**3.6 (1.0, 6.1)**	**0.006**	3.1 (0.2, 6.0)	0.04
Medication coverage				
Private insurance	Ref		Ref	
No private insurance	0.5 (-2.3, 3.2)	0.73	-0.2 (-3.0, 2.7)	0.90
Bacterial STI at baseline				
No	Ref			
Yes	**6.2 (1.9, 10.6)**	**0.006**		
Type of sexual exposure				
Condomless insertive anal	Ref			
Condomless receptive anal	**5.1 (2.6, 7.6)**	**<0.0001**		
Experienced homophobia—lifetime	**3.7 (1.4, 6.1)**	**0.002**	**3.0 (0.1, 5.9)**	**0.04**
Experienced homophobia—often	1.5 (-1.6, 4.4)	0.35		
DCHR scale	**0.2 (0.1, 0.3)**	**0.002**		
MAGI-MSV scale	0.0 (-0.2, 0.1)	0.93		
Benefits of BarebackingScale	**0.2 (0.1, 0.3)**	**0.002**	0.1 (-0.1, 0.3)	0.19
Sexual Sensation Seeking Scale	**0.4 (0.2, 0.7)**	**0.0004**	0.1 (-0.2, 0.5)	0.36

In our final linear regression model, we found that neither of the individual syndemic conditions in isolation, nor any of the 2-way, 3-way or 4-way interaction terms between these conditions, was associated with the HIRI-MSM score (data not shown). Taken together with the analyses in Table 4, this finding suggests that the relationship between syndemic conditions and HIV risk in this cohort was additive rather than synergistic in nature.

## Discussion

Our findings suggest an alarmingly high burden of syndemic health problems in nPEP patients (74%), with high proportions screening positive for depression (54%), harmful alcohol use (35%), problematic drug use (30%), and sexual compulsivity (16%). In exploratory analyses, we further observed positive associations between depression and harmful alcohol use, and between harmful alcohol use and problematic drug use, suggesting that syndemic conditions frequently cluster together in individuals. Finally, we found that the total number of syndemic conditions in a given individual was associated with increasing HIV risk, as estimated using the HIRI-MSM risk score, but that these conditions had an additive, rather than truly synergistic effect.

Our findings mirror the high levels of syndemic conditions we have observed in MSM using or considering HIV pre-exposure prophylaxis (PrEP),[32] and are in stark contrast to rates in the general population. For example, the Canadian Community Health Survey-Mental Health reports that only 4.7%, 3.2% and 0.7% of Canadians meet criteria for depression, alcohol dependence/abuse, and drug abuse during the previous 12 months, respectively, albeit using different assessment instruments.[33] Rates of sexually compulsive behavior in the general population are similarly low at 3–6%,[34] although rates among MSM have been up to 19.3%.[15]

To our knowledge, only a single other study has previously examined the burden of syndemic conditions among nPEP patients, in a cohort of 894 adults in Boston.[17] The authors found that the prevalence of pre-existing mental health conditions was high at 40.0%, including depression (24.4%), anxiety (21.9%), substance use disorders (14.4%), attention deficit disorder (7.8%), post-traumatic stress disorder (3.3%) and psychotic disorders (3.3%). Of note, that study classified participants as having mental health issues based on medication use and/or referrals for treatment, which may have underestimated the prevalence of conditions that were undiagnosed. The estimated prevalence of depression and substance use problems in our study was considerably higher, although we used screening tools with limited specificity rather than formal diagnostic tests. Taken together, however, these findings suggest that interrelated syndemic conditions are common among MSM using nPEP, and that measures to systematically assess and provide onward referrals for them may be warranted in this population.

Such assessments are important for at least two reasons. First, these syndemic conditions are associated with significant morbidity and mortality in their own right, and it is ethically and clinically important to link patients with unmet health needs into appropriate care. Second, previous literature has shown that these syndemic conditions are strongly associated with HIV risk behaviours, prevalent HIV infection, and most importantly, incident HIV infection.[11–15, 35, 36] Our finding of a positive, statistically significant relationship between syndemic count and HIV risk (as measured by the HIRI-MSM score) further supports this contention. By identifying these issues and referring affected patients into care, it is hoped that HIV risk could be reduced over the longer term.

Of interest, a randomized trial is currently underway in Amsterdam among MSM with high risk sexual behaviour to test whether administering and providing feedback on questionnaires about a variety of syndemic domains (including depression, anxiety, sex- and drug addiction) increases help-seeking behaviour for those very problems.[37] Whether such interventions could further lead to improvements in risk behaviours and HIV infections remains unknown, and empirical data on their impact on such downstream outcomes are greatly needed.

Syndemic theory posits that the individual conditions not only predict future adverse outcomes, but that they are clustered, and mutually reinforcing. In our cohort of nPEP patients, as in the above-mentioned Boston cohort,[17] there were several positive bivariable associations between the individual syndemic conditions observed. However, an interesting point of contention in the literature is whether the effects of individual conditions on HIV risk among MSM are truly multiplicative (implying synergy) or simply additive in nature.[38] The original theoretical description and etymologic origin of the term ‘syndemics’ explicitly refer to synergy between co-occurring epidemics, that combine to produce an excess burden of disease.[39] However, most studies have instead found an additive effect,[11–15] and a systematic review of studies employing the syndemic framework to understand HIV risk found that only 28% of studies formally tested for multiplicative effects.[38] That we observed a relationship between syndemic count and HIV risk, but not between any interactions between these conditions and HIV risk, supports an additive rather than multiplicative model. However, we caution that our analyses were exploratory in nature, and we concur with other authors who argue that the development of interventions must not wait for the methodologic question of interaction to be resolved.[40]

In addition to syndemic count, we observed that lifetime experience of homophobia was also associated with higher HIRI-MSM scores in our exploratory multivariable model. This finding is consistent with another tenet of syndemic theory, which situates these interrelated health challenges within a life-course of social marginalization.[9, 41, 42] Prior literature has proposed that experiences of discrimination contribute to the emergence of syndemic health problems both directly,[43–45] and/or mediated through internalized homophobia.[46] Several potential mechanisms for these relationships have been described, including emotional dysregulation, anxiety, and depression.[13, 46–48] Despite low levels of internalized homophobia in our cohort (represented by low MAGI-MSV scores), the overall levels of self-reported lifetime discrimination were high (59%). These findings underscore the role of broader social forces in driving health outcomes in MSM, as abundantly illustrated in literature from diverse global settings,[49, 50] and highlight the need for advocacy and structural change as part of a combination HIV prevention approach. Examples of structural interventions that could reduce stigma and discrimination may be legislative (eg. expansion of civil rights), programmatic (eg. providing queer-friendly services) and social (eg. public efforts to decrease homophobia). While significant strides have been made in this regard in Canada (eg. legalization of same-sex marriage nationwide in 2005, participation of the sitting Prime Minister in gay pride parades for the first time in 2016), efforts to address anti-gay discrimination must be sustained and expanded.

The only other variable associated with HIV risk in our multivariable model was moderate/high perceived HIV risk. This finding may reflect some degree of participant awareness of their own risk, and is encouraging because such awareness is an important first step in being receptive to modifying this risk. However, 57% of participants perceived their lifetime HIV risk to be either “no risk” or “low risk”, despite all presenting for sexual exposures that were high enough risk to warrant nPEP. In addition, respondents’ low overall levels of perceived risk contrast with their relatively high levels of objective HIV risk as measured by the HIRI-MSM, with 81% meeting the cutoff for high risk.[18] This disparity parallels the misconceptions regarding HIV risk that we and others have observed in studies among MSM.[51–55] Clinical encounters for nPEP may be an ideal setting in which to counsel patients in greater detail about how they perceive their HIV risk, given that these patients have identified themselves as being concerned about this issue. Patients presenting for nPEP may further be an ideal population in which to test HIV risk reduction interventions grounded in syndemic theory, for the same reason. In this regard, it is also noteworthy that overall levels of HIV fatalism and optimism in our study cohort were relatively low, even though HIV-related anxiety was moderately high. These findings suggest that participants were not heavily reliant on ‘disengagement coping’ strategies (ie. maladaptive responses to the stress of HIV risk including fatalistic attitudes and HIV-related anxiety), and again may suggest that nPEP patients may be receptive to syndemics-based risk reduction interventions.

This study has limitations that warrant consideration. First, the tools used to measure syndemic conditions in this study were self-administered screening questionnaires and therefore do not directly represent clinical diagnoses; further, the stress of being recently exposed to HIV may have biased participants’ questionnaires towards more pessimistic responses. Nevertheless, these tools may still be useful to screen for patients warranting further diagnostic evaluation. Second, the cohort was highly educated, and we restricted analyses to MSM, limiting our ability to draw conclusions about other groups. However, this sample does reflect the population typically seen for nPEP in our setting,[56, 57] and likely reflects the success of awareness-raising efforts in MSM communities. Of note, people who used nPEP in the context of sexual assault were unlikely included in this sample, since these patients are usually seen at sexual assault centres separate from our hospitals. Third, only 203/375 individuals approached about the study agreed to participate. Although the reasons for non-participation were not recorded prospectively, the most common reasons were a lack of time and a preoccupation with the need for PEP, and it is unclear in what direction the omission of these individuals may have biased our findings. Fourth, the HIRI-MSM tool which we used to estimate HIV risk has intrinsic limitations related to the 1990s dataset from which it was derived.[58] In particular, the scale does not consider the tremendous impact of suppressive antiretroviral therapy on preventing HIV transmission,[59, 60] and thus may overestimate risk, although higher scores should still represent higher risk overall. Lastly, our study was not powered to quantify the relationships between syndemic health problems and HIV risk, and our logistic regression analyses should thus be considered exploratory in nature. Because comprehensive screening of all nPEP patients for syndemic problems may not be practical in all settings, larger studies should examine these issues in the future to develop more targeted approaches.

In summary, we suggest that nPEP clinical encounters be harnessed as an opportunity to routinely screen for syndemic health problems among MSM, and that affected patients be linked to related services as appropriate. Such a strategy exemplifies the concept of ‘combination HIV prevention’,[61–63] which posits that biological, behavioural, and psychosocial problems should be addressed in combination for effective HIV prevention. The success of such a screening program in reducing HIV risk would be contingent on factors including physician uptake, patient willingness to be screened, availability and efficacy of treatment for syndemic problems, and the downstream impact of treating syndemics on HIV acquisition. Further work should be done to elucidate the optimal program structure, feasibility, effectiveness and efficiency at decreasing long-term HIV risk.
